# Supplementary material for: Evaluation of Nutritional Quality and Oxidation Stability of Fermented Edible Insects
Source: Foods. 2025 Aug 22;14(17):2929. doi: 10.3390/foods14172929 (PMC12428198; doi:10.3390/foods14172929)
Supplement: Supplementary file 1 [file foods-14-02929-s001.zip › foods-3799463-supplementary.pdf]

Supplementary material

Evaluation of Nutritional Quality and Oxidation Stability of Fermented Edible Insects

Anja Vehar[1,2], Doris Potočnik[1], Marjeta Mencin[1], Mojca Korošec[3], Blaž Ferjančič[3], Marta Jagodic Hudobivnik[1], Polona Jamnik[3], Ajda Ota[4], Lenka Kouřimská[5], Martin Kulma[6], David Heath[1], Nives Ogrinc[1,2]

S1. Appendix to methods

Table S1.Monitored ions for amino acid analysis

Amino acid	t_R [min]	Quantifier	Qualifier1	Qualifier2
alanine	1.389	130	88	116
glycine	1.503	116	102	74
valine	1.716	158	116	72
leucine	1.938	172	86	116
isoleucine	1.995	172	130	101
threonine	2.206	101	143	203
4-aminobutanoic acid	2.248	144	130	112
serine	2.248	146	101	60
proline	2.332	156	70	114
aspartic acid	2.999	216	88	130
methionine	3.032	203	101	277
hydroxyproline	3.18	172	86	68
glutamic acid	3.369	230	84	170
phenylalanine	3.397	148	190	120
lysine	4.713	170	128	84
histidine	4.904	282	266	168
hydroxylysine	5.081	129	169	116
tyrosine	5.19	107	206	164

S2. Statistical analysis

Table S2. Significant differences in all measured parameters between all species and yellow mealworm vs. house cricket for nutritional composition (= $p < 0.05$, x = not statistically analyzed)*

	All species	YM vs. HC		All species	YM vs. HC
alanine	*	x	Induction time		x
glycine		x	Dry matter	x	
valine	*	x	Water	x	
leucine	*	x	Ash	x	*
isoleucine	*	x	Total fat	x	
threonine		x	Insoluble dietary fiber	x	
serine	*	x	Soluble dietary fiber	x	
proline	*	x	Total dietary fiber	x	
aspartic acid	*	x	Total nitrogen	x	*

methionine	*	x	Non-protein nitrogen	x	
glutamic acid		x	Protein nitrogen	x	
phenylalanine	*	x	Proteins	x	
ornithine	*	x	Available carbohydrates	x	x
lysine	*	x	Energy value	x	x
histidine	*	x	B	*	x
tyrosine	*	x	Na		x
Total EAA		x	Mg	*	x
Total AA		x	Al	*	x
C6:0	x	x	P	*	x
C10:0	*	x	S	*	x
C12:0	*	x	K	*	x
C13:0	*	x	Ca	*	x
C14:0	*	x	V	*	x
C14:1	*	x	Mn	*	x
C15:0	*	x	Ag	*	x
C16:0	*	x	Cd	*	x
C16:1	*	x	Sn	*	x
C17:0	*	x	Sb	*	x
C17:1	*	x	Cs	*	x
C18:0	*	x	Ba	*	x
C18:1 cis 9	*	x	Hg	*	x
C18:2 cis 9,12	*	x	Pb	*	x
C20:0		x	U	*	x
C20:1 cis 11		x	Fe	*	x
C18:3 cis 9,12,15 alpha	*	x	Co	*	x
C20:2 cis 11,14	*	x	Ni		x
C22:0	*	x	Cu	*	x
C24:0		x	Zn		x
C24:1 cis 15	*	x	As	*	x
SFA	*	x	Se	*	x
MUFA	*	x	Rb	*	x
PUFA	*	x	Sr	*	x
n-6	*	x	Mo	*	x
n-3	*	x	Daily intake Ca	*	x
n-6/n-3	*	x	Daily intake Cu	*	x
IA	*	x	Daily intake Fe	*	x
IT	*	x	Daily intake K	*	x
h/H	*	x	Daily intake Mg	*	x
			Daily intake Mn	*	x
			Daily intake Na		x
			Daily intake P	*	x
			Daily intake Se	*	x
			Daily intake Zn		x

S3. Fatty acids analysis

Table S3. Fatty acid profile (%)

	omega	saturation	YM	YMP	YMM	HC	HCP	HCM	ML	MLP	MLM
hexanoic acid		SFA	0 ± 0	0 ± 0	0 ± 0	0 ± 0	0 ± 0	0 ± 0	0 ± 0	0.030 ± 0.000	0.033 ± 0.001
capric acid		SFA	0.015 ± 0.000	0.014 ± 0.000	0.014 ± 0.000	0 ± 0	0 ± 0	0 ± 0	0.020 ± 0.000	0.023 ± 0.000	0.010 ± 0.015
lauric acid		SFA	0.340 ± 0.002	0.327 ± 0.003	0.328 ± 0.000	0.044 ± 0.001	0.048 ± 0.001	0.047 ± 0.000	0.179 ± 0.003	0.188 ± 0.000	0.185 ± 0.001
tridecanoic		SFA	0.071 ± 0.000	0.071 ± 0.001	0.071 ± 0.000	0.002 ± 0.002	0.004 ± 0.000	0 ± 0	0 ± 0	0 ± 0	0 ± 0
myristic acid		SFA	3.79 ± 0.02	3.76 ± 0.05	3.76 ± 0.01	0.504 ± 0.005	0.533 ± 0.006	0.525 ± 0.003	1.29 ± 0.01	1.41 ± 0.00	1.41 ± 0.01
myristoleic acid		MUFA	0.023 ± 0.002	0.022 ± 0.001	0.022 ± 0.001	0.024 ± 0.002	0.027 ± 0.001	0.026 ± 0.001	0.202 ± 0.006	0.174 ± 0.004	0.184 ± 0.005
pentadecanoic acid		SFA	0.117 ± 0.000	0.121 ± 0.001	0.120 ± 0.000	0.087 ± 0.001	0.092 ± 0.001	0.091 ± 0.000	0.091 ± 0.010	0.074 ± 0.000	0.077 ± 0.005
palmitic acid		SFA	16.0 ± 0	16.2 ± 0.2	16.2 ± 0.1	23.8 ± 0.2	25.0 ± 0.1	24.7 ± 0.2	19.8 ± 0.1	20.9 ± 0.0	20.9 ± 0.1
palmitoleic acid		MUFA	1.68 ± 0.00	1.67 ± 0.00	1.67 ± 0.00	0.763 ± 0.013	0.795 ± 0.003	0.775 ± 0.002	0.805 ± 0.013	0.801 ± 0.002	0.813 ± 0.019
margaric acid		SFA	0.148 ± 0.006	0.153 ± 0.002	0.152 ± 0.000	0.155 ± 0.002	0.161 ± 0.000	0.158 ± 0.000	0.353 ± 0.002	0.309 ± 0.001	0.318 ± 0.018
cis-9-Heptadecenoic acid		MUFA	0.118 ± 0.001	0.125 ± 0.002	0.121 ± 0.003	0.061 ± 0.005	0.017 ± 0.012	0.059 ± 0.006	0.113 ± 0.031	0.11 ± 0.00	0.112 ± 0.015
stearic acid		SFA	3.20 ± 0.00	3.35 ± 0.03	3.34 ± 0.01	8.61 ± 0.01	8.81 ± 0.02	8.81 ± 0.09	9.59 ± 0.03	9.83 ± 0.04	9.87 ± 0.04
oleic acid		MUFA	46.5 ± 0.0	46.0 ± 0.3	46.0 ± 0.1	31.9 ± 0.5	31.4 ± 0.0	31.6 ± 0.1	28.2 ± 0.3	28.0 ± 0.0	27.9 ± 0.0
linoleic acid	6	PUFA	26.4 ± 0.0	26.5 ± 0.0	26.5 ± 0.0	31.4 ± 0.1	30.8 ± 0.0	30.9 ± 0.2	29.8 ± 0.2	28.4 ± 0.0	28.5 ± 0.0
arachidic acid		SFA	0.126 ± 0.001	0.295 ± 0.000	0.287 ± 0.001	0.194 ± 0.002	0.212 ± 0.001	0.210 ± 0.001	0.214 ± 0.001	0.268 ± 0.003	0.271 ± 0.001
cis-11-Eicosenoic acid		MUFA	0.137 ± 0.000	0.144 ± 0.002	0.143 ± 0.001	0.114 ± 0.003	0.117 ± 0.003	0.113 ± 0.001	0 ± 0	0.171 ± 0.000	0.171 ± 0.002
α-linolenic acid	3	PUFA	1.00 ± 0.01	0.959 ± 0.002	0.968 ± 0.000	1.85 ± 0.01	1.77 ± 0.01	1.77 ± 0.01	9.06 ± 0.06	9.06 ± 0.00	9.10 ± 0.00
eicosadienoic acid	6	PUFA	0.190 ± 0.001	0.203 ± 0.001	0.204 ± 0.000	0.179 ± 0.002	0.063 ± 0.012	0.051 ± 0.008	0.090 ± 0.004	0.041 ± 0.002	0.040 ± 0.056
behenic acid		SFA	0.095 ± 0.001	0.117 ± 0.009	0.096 ± 0.003	0.025 ± 0.000	0.025 ± 0.005	0.019 ± 0.002	0.034 ± 0.000	0.050 ± 0.001	0.025 ± 0.035
lignoceric acid		SFA	0.018 ± 0.000	0.020 ± 0.000	0.024 ± 0.000	0.186 ± 0.242	0.071 ± 0.051	0.053 ± 0.009	0.081 ± 0.004	0 ± 0	0 ± 0

S4. Risk assessment

Table S4. Reference values for risk assessment

**Al**	EFSA 2018	ADI	mg/(kg b.w. day)	0.14
**As (inorganic derivatives)**	EFSA CONTAM, 2009	PTWI	µg/(kg b.w. week)	1.5
**Ca**	EFSA ANS 2012	UL	mg/day	2500
**Cd**	EFSA CONTAM 2011	TWI	µg/(kg b.w. week)	2.5
**Co (derivatives)**	EFSA FEEDAP 2009	ADI	mg/(kg b.w. day)	600
**Fe**	EFSA 2024	SI	mg/day	40
**Hg**	EFSA CONTAM 2005	TWI	µg/(kg b.w. week)	1.6
**Ni**	EFSA CONTAM 2020	TDI	µg/(kg b.w. day)	13
**Pb**	EFSA CONTAM 2005	TWI	µg/(kg b.w. week)	2.5
**Se**	EFSA FEEDAP 2016	UL	µg/day	300
**U**	EFSA CONTAM 2009	TDI	g/(kg b.w. day)	0.6
**Zn**	EFSA FEEDAP 2017	UL	mg/day	25

ADI – acceptable daily intake, PTWI – provisional tolerable weekly intake, SI – safe level of intake, TDI – tolerable daily intake, TWI – tolerable weekly intake, UL – tolerable upper intake level

Table S5. Population reference intake and adequate intake values

**Ca**	EFSA NDA 2015	PRI	mg/day	1000
**Cu**	EFSA NDA 2015	AI	mg/day	1.3
**Fe**	EFSA NDA 2015	PRI	mg/day	16
**K**	EFSA NDA 2016	AI	mg/day	3500
**Mg**	EFSA NDA 2015	AI	mg/day	300*
**Mn**	EFSA NDA 2013	AI	mg/day	3
**Na**	EFSA NDA 2019	AI	mg/day	2000
**P**	EFSA NDA 2015	AI	mg/day	500

**Se**	EFSA CEF 2018	AI	mg/day	0.07
**Zn**	EFSA NFA 2014	PRI	mg/day	11**

*for women, 350 for men

** PRI ranges from 7.5 to 16.3, so mid-range PRI was used

PRI – population reference intake, AI – adequate intake

Table S6. Intake values calculated for the consumption of 100 g of fresh insects for risk assessment

Element	Unit	YM	YMP	YMM	HC	HCP	HCM	ML	MLP	MLM
Al	mg/(kg b.w. day)	8.66E-04	1.15E-03	9.23E-04	3.56E-02	2.45E-02	2.54E-02	3.33E-02	5.37E-02	3.87E-02
As	µg/(kg b.w. week)	0.154	0.161	0.160	0.048	0.051	0.040	0.070	0.076	0.194
Ca	mg/day	15.5	14.8	15.4	37.9	36.8	41.4	27.1	27.7	30.5
Cd	µg/(kg b.w. week)	0.256	0.298	0.291	0.112	0.098	0.095	0.127	0.155	0.112
Co	mg/(kg b.w. day)	2.50E-05	2.58E-05	2.47E-05	3.39E-05	2.91E-05	3.08E-05	1.23E-05	1.52E-05	1.47E-05
Fe	mg/day	1.38	1.29	1.30	1.74	1.58	1.84	1.57	1.93	1.87
Hg	µg/(kg b.w. week)	2.82E-03	3.26E-03	3.21E-03	6.41E-03	5.16E-03	6.17E-03	1.06E-02	1.24E-02	9.73E-03
Ni	µg/(kg b.w. day)	0.210	0.267	0.228	0.092	0.106	0.102	0.365	0.336	0.363
Pb	µg/(kg b.w. week)	0.0216	0.0234	0.0207	0.333	0.263	0.281	0.314	0.432	0.340
Se	µg/day	4.24	4.54	4.36	9.80	9.94	9.52	8.40	7.07	8.89
U	g/(kg b.w. day)	3.38E-08	4.03E-08	3.74E-08	4.51E-08	4.05E-08	3.97E-08	1.84E-09	2.22E-09	1.73E-09
Zn	mg/day	4.00	3.93	4.04	7.00	6.02	6.17	3.56	3.54	3.98
